# An Unusual Presentation of Wilson's Disease

**DOI:** 10.7759/cureus.58407

**Published:** 2024-04-16

**Authors:** Shubhangi Kanitkar, Akshata Borle, Muskaan Ahlawat, Sai Priya Ande, Sandesh Raut

**Affiliations:** 1 Internal Medicine, Dr. D. Y. Patil Medical College, Hospital and Research Centre, Pune, IND

**Keywords:** cirrhosis, young child, copper metabolism, kayser fleischer ring, wilson disease

## Abstract

Wilson's disease affects the metabolism of copper and is a rare hereditary disorder that is inherited autosomally recessively. The liver and brain are the main organs affected by this disorder, which causes progressive hepatolenticular degeneration. A 15-year-old male patient arrived at the outpatient department (OPD) with mild abdominal pain on the right side, and both eyes showed Kayser-Fleischer (KF) rings. An abdominal ultrasound showed that the spleen was enlarged. Copper levels in urine were found to be higher. After a liver biopsy, cirrhosis, and mild chronic active hepatitis were found. The majority of hematological indicators were normal; however, a peripheral blood smear revealed mild thrombocytopenia. Wilson's disease is uncommon, so diagnosing it requires a high degree of suspicion. In circumstances of inexplicable liver cirrhosis or isolated neurological symptoms, it could go unnoticed. The only primary complaint in the case being presented was abdominal pain. However, the age upon presentation, the existence of KF rings in both eyes, and other tests helped us get the diagnosis.

## Introduction

Wilson's disease is a rare hereditary disorder affecting the metabolism of copper, inherited autosomally recessively. Neurological and hepatic impairment are the primary symptoms [1]. An estimated one in 30,000 live births are affected by Wilson's disease annually, with the East Asian region having a significantly higher prevalence [2,3]. Chromosome 13's ATP7B gene mutation causes an incorrect copper transport protein to be produced. This genetic defect results in an accumulation of copper in many tissues. The brain and liver are commonly involved. Neurological symptoms include changes in behavior, tremors, slurring, and drooling. Hepatic symptoms include fatigue, edema, nausea, vomiting, loss of appetite, jaundice, and easy bruising. Patients may also develop corneal Kayser-Fleischer (KF) rings. Mutations in one copy of the gene ATP7B are carriers of Wilson's disease. People with Wilson's disease have mutations in both copies of the gene [4-7]. Wilson's disease carriers do not need treatment, but those who have the illness, symptoms or not, need lifelong care.

## Case presentation

A 15-year-old male patient came to the outpatient department complaining of sporadic right-sided abdominal pain. There was no history of fever, altered bowel habits, nausea or vomiting, or significant weight loss. There was no notable family history. Pallor, icterus, cyanosis, clubbing, lymphadenopathy, and edema were not present on general examination. During the examination of the abdomen, the spleen was felt halfway between the left costal border and the umbilicus indicating splenomegaly. Neurological examination was normal. Laboratory investigations and work-up for Wilson's disease were sent (Tables 1, 2).

**Table 1:** Laboratory investigations on presentation EDTA: Ethylenediaminetetraacetic acid, HDL: High-density cholesterol, LDL: Low-density cholesterol, VLDL: Very low-density cholesterol, INR: International Normalized Ratio, HIV: Human Immunodeficiency Virus, HCV: Hepatitis C virus, HBsAg: Hepatitis B antigen

Parameters	Patient values	Normal range
Complete blood count		
Hemoglobin (Hb), EDTA whole blood	11.50 g/dl	12.8 - 16 g/dl
Total leucocyte count	4,900/mcL	4000 - 9100/mcL
Platelet count	1,43,000/mcL	150000 - 410000/mcL
Red blood cell count	4.85 x 10^6 /mcL	4.4 - 5.5 x 10^6 /mcL
Hematocrit	36.70 %	37.3 - 47.3 %
Mean corpuscular volume	75.7 fL	81.4 - 91.9 fL
Mean corpuscular hemoglobin	23.70 pgms	27.0 - 32.0 pgms
Mean corpuscular hemoglobin concentration	31.40 g/dl	31.5 - 34.5 g/dL
RBC distribution width	15.30%	11.6 - 13.8%
Mean platelet volume	8.1 fL	7.4 - 11.4 fL
Glycosylated Hb	5%	4.0 to 5.6 %
WBC differential count		
Neutrophils	45%	
Absolute neutrophils	2,205/mcL	1800 - 8000/mcL
Eosinophils	3%	
Absolute eosinophils	147/mcL	0 - 500/mcL
Basophils	0%	
Absolute Basophils	0/mcL	0 - 200/mcL
Lymphocytes	42%	
Absolute lymphocytes	2,058/mcL	1200 - 5200/mcL
Monocytes	10%	
Absolute monocytes	490/mcL	0-800/μL
Coagulation profile		
Prothrombin time	14.90 secs	10.09 - 13.79 secs
INR	1.25	0.85 - 1.15
Lipid profile		
Serum total cholesterol	148 mg/dL	< 200 mg/dL
Serum triglycerides	54 mg/dL	< 150 mg/dL
Serum HDL	43 mg/dL	> 40 mg/dL
Serum VLDL	11 mg/dL	< 30 mg/dL
Serum LDL	94 mg/dL	<100 mg/dL
HIV, HBsAg, HCV	Non-reactive	

**Table 2:** Wilson's disease work-up and routine investigations SGOT: Serum glutamic oxaloacetic transaminase, SGPT: Serum glutamic pyretic transaminase, TSH: Thyroid stimulating hormone, T3: Triiodothyronine

	Values	Normal range
Liver function test		
Total serum bilirubin	0.49 mg/dL	0.22 - 1.20 mg/dL
Conjugated serum bilirubin	0.25 mg/dL	Upto 0.5 mg/dL
Unconjugated serum bilirubin	0.24 mg/dL	0.1 to 1.0 mg/dL
SGOT	36 U/L	8 - 48 U/L
SGPT	24 U/L	7 to 55 U/L
Alkaline phosphatase	194 U/L	82 - 331 U/L
Proteins in serum		
Total serum protein	7.6 g/dL	6.4 to 8.3 g/dL
Serum albumin	4.3 g/dL	3.5 to 5.2 g /dL
Serum globulin	3.3 g/dL	2.3 - 3.5 g/dL
Albumin-globulin ratio	1.3 g/dL	
Hormones		
Serum TSH	1.01 μIU/mL	0.50 - 4.30 μIU/mL
Serum T3	1.64 ng/ml	0.98 - 1.76 ng/ml
Serum parathormone	43.5 pg/mL	15 - 65 pg/mL
Urine routine		
Physical		
Appearance	Clear	
Color	Straw	
Deposits	Absent	Absent
Specific gravity (1.003 to 1.035)	1.010	1.003 to 1.035
Chemical		
pH	7.5	4.6 - 8.0
Protein	Traces	Absent
Glucose, acetone, bile pigments	Absent	Absent
Microscopy		
RBCs	1-2/hpf	0 - 2/hpf
Pus cells	1-2/hpf	0 - 5/hpf
Epithelial cells	1-2/hpf	0 - 5/hpf
Magnesium, serum by calmagite dye	1.90	1.8 - 2.4 mg/dl
Calcium serum by Arsenazo III method	9.30	8.6 - 10.2 mg/dl
Phosphorus, serum by ammonium molybdate UV	5.50	2.6 - 4.7
24 hours urine copper	394.45 mcg/24 hours	20 - 50 mcg/24 hours
Serum copper	53 microgram/dL	63.5 - 150 microgram/dL
Serum ceruloplasmin	0.03	0.22 - 0.58 g/L

**Figure 1:**
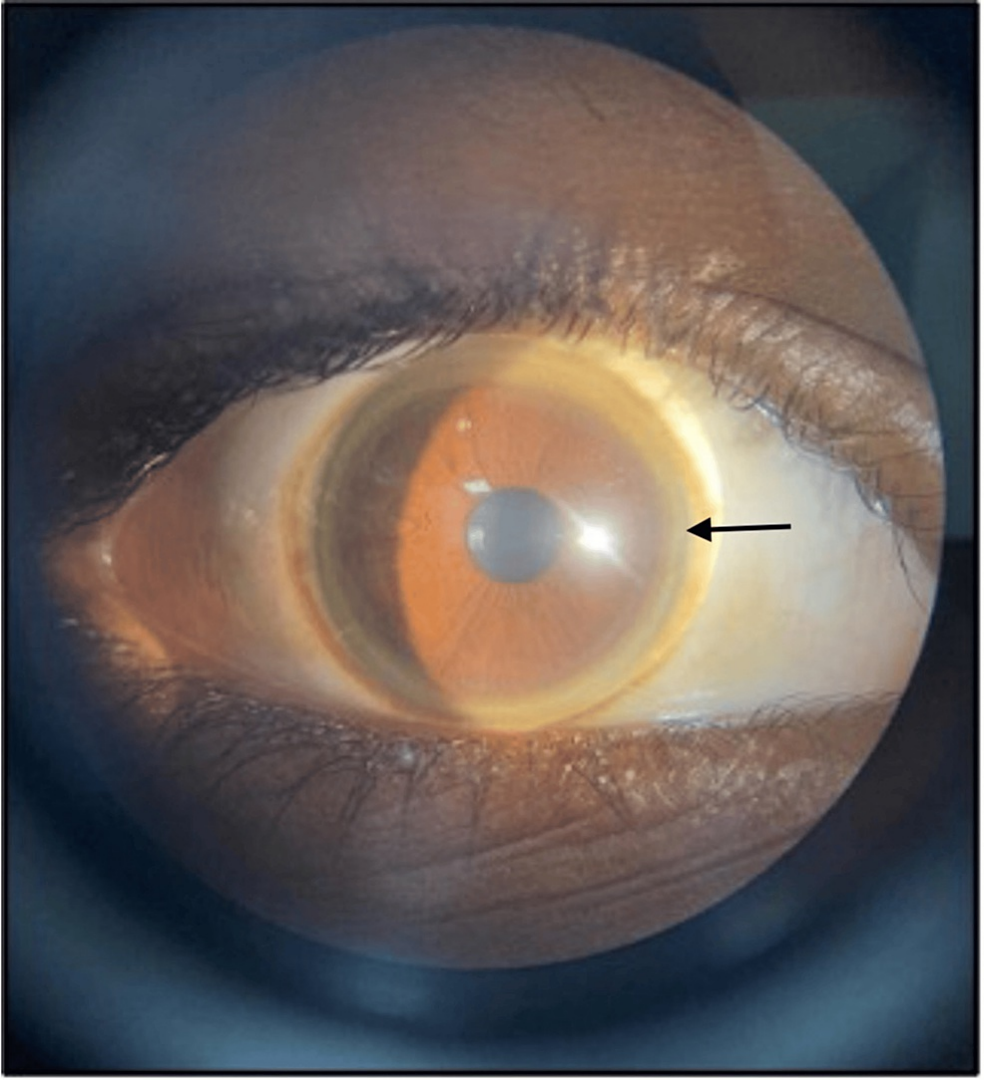
Kayser-Fleischer ring as seen on slit lamp The black arrow indicates the Kayser-Fleischer ring

The spleen measured 12.8 cm in size on abdominal ultrasound (USG). Contrast-enhanced computed tomography (CECT) of the abdomen and pelvis showed cirrhotic changes in the liver and splenomegaly. The increased copper levels in the urine and presence of KF rings on slit lamp examination were suggestive of Wilson's disease (Figure 1)

**Figure 2:**
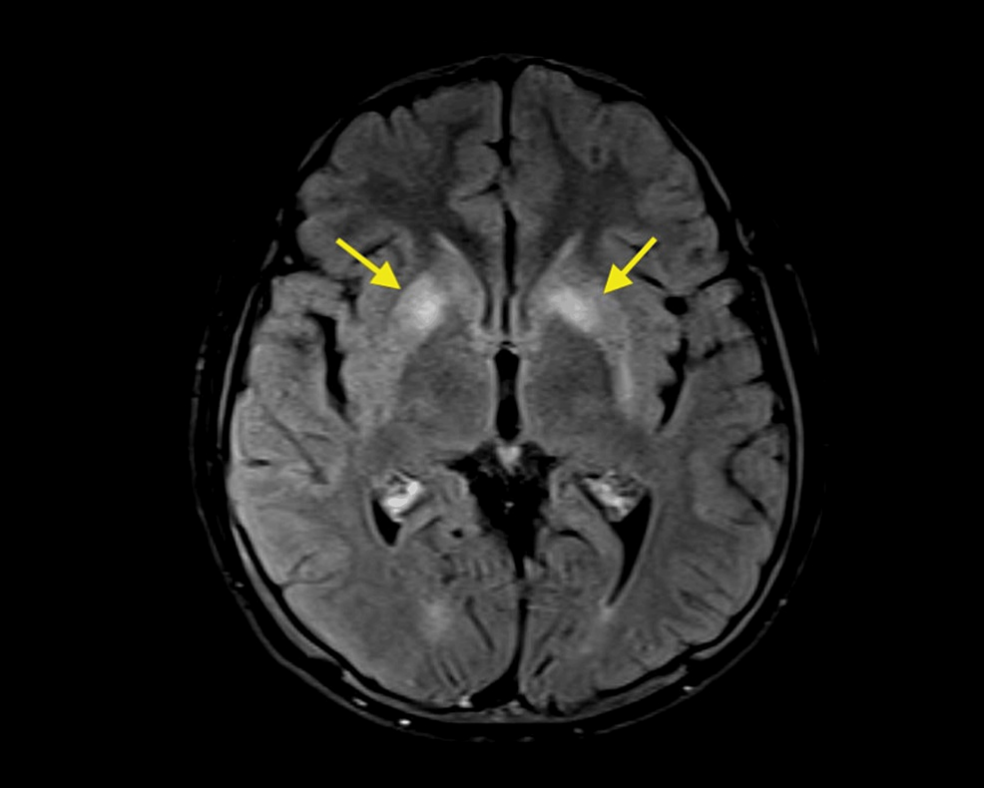
MRI STIR transverse section of the brain Yellow block arrows show hyperintensity in the bilateral ganglio-capsular region MRI: Magnetic resonance imaging, STIR: Short tau inversion recovery

MRI brain (plain and contrast) showed multiple altered signal intensity areas in bilateral ganglio-capsular regions involving deep grey matter (Figure 2).

**Figure 3:**
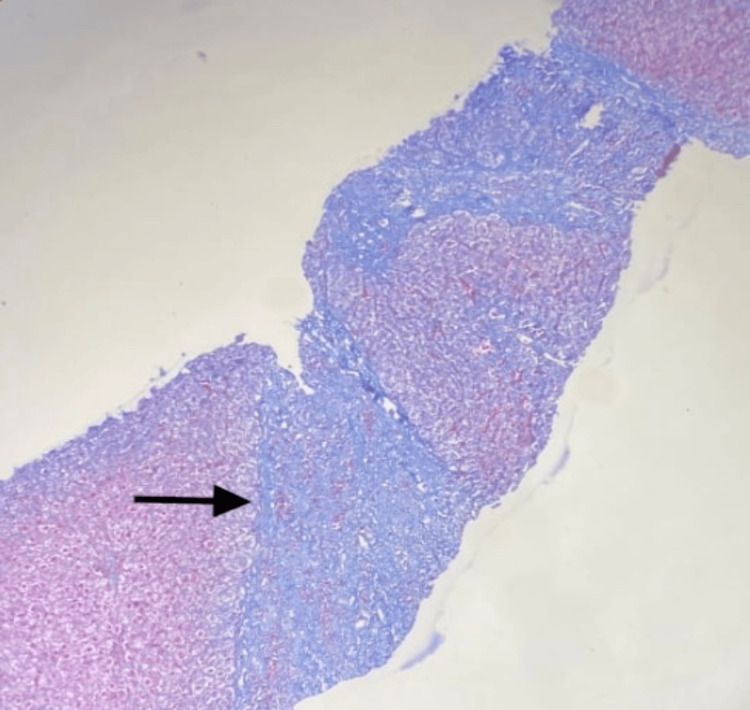
Nodules of varying sizes separated by fibrous septae and inflammatory infiltrate (100 X, low power) The black arrow indicates the fibrous septae

Based on preliminary findings, a provisional diagnosis of Wilson's was made. To confirm the diagnosis we did a liver biopsy. According to the histological results of the liver sample, there was mild chronic active hepatitis along with cirrhosis of the liver, stage 4 fibrosis according to Batts-Ludwig staging, and grade 2 inflammation. Liver biopsy copper analysis showed copper content of 62 microgram/gm (normal range is up to 45 microgram/gm). Figure 3 shows fibrosis and mild chronic active hepatitis suggesting chronic histopathological changes in Wilson's disease. 

## Discussion

Wilson's disease patients may exhibit slowly progressing cirrhosis, which is typically well compensated. There might be a severe illness (acute liver failure) without any neurological symptoms. Measurements of the liver's copper level in conjunction with a liver biopsy may aid in the diagnosis. Once diagnosis was established, D-penicillamine therapy was initiated. Initially, D-penicillamine 250 mg once a day (OD) was started followed by 250 mg increments every four days.

Similar cases, mostly involving individuals over 40, have been reported [8,9], where the first presentation is related to the neurological system without hepatic involvement. 

The mobilization of copper from the liver, which raises levels of unbound copper and exacerbates neurological symptoms, is assumed to be the cause of this deterioration. The frequency of neurological damage following penicillamine therapy has been the subject of numerous studies. While some research cast doubt on this association, others claim that rates range from 30 to 75% of patients [10,11]. Improving the patient's condition is the aim of the initial phase of treatment. The first-line medications are penicillamine and trientine. Penicillamine increases urine excretion to as much as 15-45 μmol daily and chelates copper. Penicillamine may cause the neurological symptoms to worsen. Patients who are intolerant to D-penicillamine are administered trientine. For adults, 1-3 g of D-penicillamine orally three to four times a day, one hour prior to meals is given. Children's dosage is 20 mg/kg, administered twice daily. When administered gradually, starting at a dose of 250 mg once daily and increasing by another 250 mg every four to five days until the full daily dose is reached, penicillamine is frequently better tolerated by patients. It is advised to maintain medication at this dose for at least six months because of the slow rate of improvement. One way to handle maintenance is to lower the chelator dose or replace it with zinc. Zinc restricts the absorption of copper via the gastrointestinal system by inducing intestinal metallothionein. Adults should take 50 mg of elemental zinc orally three times a day [12]. If combination therapy (chelator plus zinc) is used, individuals with severe neurological manifestations or decompensated hepatic disease should be the only ones to receive it, per a specialist's recommendation. For all young adults and children with chronic liver disease who do not show any symptoms, Wilson's disease should be taken into consideration. First-degree relatives of the affected individual must undergo screening for Wilson's disease.

Wilson's disease patients usually present with hepatic symptoms at first, though neurological symptoms can potentially appear later. However, in this particular instance, pain in the abdomen was the only symptom [12]. Patients should be advised not to eat foods high in copper, like shellfish, almonds, chocolate, liver, and mushrooms. Liver transplantation is required for patients with Wilson's disease-related acute liver failure, cirrhosis, and decompensated liver disease who do not get better after two to three months of medication therapy. If neglected, Wilson's illness worsens and eventually proves fatal. The patient dying without receiving treatment and going undiagnosed is the biggest risk. Individuals who are diagnosed early and adhere strictly to their treatment plan should expect to enjoy normal lives. 

## Conclusions

Wilson's illness is an uncommon condition, so it is possible that the diagnosis can go unnoticed. When isolated symptoms, like abdominal pain in a young individual, are present with liver cirrhosis without a known cause, it is imperative to maintain a high index of suspicion. It is imperative that patients be counseled against stopping the treatment for Wilson's disease. Wilson's disease prevention guidelines are still lacking. However, treatment can prevent symptoms from worsening if they are detected early.

## References

[1] Ala A, Walker AP, Ashkan K, Dooley JS, Schilsky ML (2007). Wilson's disease. Lancet.

[2] Gollan JL, Gollan TJ (1998). Wilson disease in 1998: genetic, diagnostic and therapeutic aspects. J Hepatol.

[3] Sandahl TD, Ott P (2019). Epidemiology of Wilson disease. Wilson Disease.

[4] Bull PC, Thomas GR, Rommens JM, Forbes JR, Cox DW (1993). The Wilson disease gene is a putative copper transporting P-type ATPase similar to the Menkes gene. Nat Genet.

[5] Roberts EA, Schilsky ML (2008). Diagnosis and treatment of Wilson disease: an update. Hepatology.

[6] Roberts EA, Socha P (2017). Wilson disease in children. Handb Clin Neurol.

[7] Nagral A, Sarma MS, Matthai J (2019). Wilson’s disease: Clinical practice guidelines of the Indian National Association for study of the liver, the Indian Society of Pediatric Gastroenterology, Hepatology and Nutrition, and the Movement Disorders Society of India. J Clin Exp Hepatol.

[8] Kaur H, Kaur K, Sharma N, Kumar K (2019). Wilson's disease: a case report. Int J Contemp Med Res.

[9] Ferenci P, Członkowska A, Merle U (2007). Late-onset Wilson's disease. Gastroenterology.

[10] Kalita J, Kumar V, Chandra S, Kumar B, Misra UK (2014). Worsening of Wilson disease following penicillamine therapy. Eur Neurol.

[11] Medici V, Trevisan CP, D'Incà R (2006). Diagnosis and management of Wilson's disease: results of a single center experience. J Clin Gastroenterol.

[12] Sherlock S, Dooley J (2008). Diseases of the liver and biliary system. https://www.google.co.in/books/edition/Diseases_of_the_Liver_and_Biliary_System/-TD1_-SiqvcC?hl=en&sa=X&ved=2ahUKEwjE4rumzpaFAxWvcGwGHfCwBCsQiqUDegQIEBAC.

